# DNA Methylation Profiles of Long-Term Cannabis Users in Midlife: An Evaluation of Published Markers

**DOI:** 10.1093/geroni/igaf122.1968

**Published:** 2025-12-31

**Authors:** Madeline Meier

**Affiliations:** Arizona State University, Tempe, Arizona, United States

## Abstract

Long-term cannabis use has been linked to a number of health problems, including respiratory problems, cardiovascular diseases, cognitive decline, and mental illness. Epigenetic responses to cannabis use could underlie cannabis-related health problems. We examined the DNA-methylation profiles of long-term cannabis users in midlife, re-evaluating a set of 246 previously published cannabis-associated methylation markers. Data were from the Dunedin Study, a five-decade longitudinal study of a birth cohort (analytic n = 787). Peripheral whole blood was drawn when the cohort was age 45, and DNA methylation was assayed using the EPIC 850K BeadChip. Analyses compared long-term cannabis users with non-users and, for a benchmark comparison, with long-term tobacco users. Results showed that long-term cannabis use was associated with sixteen of the previously published 246 cannabis-related methylation markers. All cannabis-related methylation markers were also associated with long-term tobacco use. Nonetheless, after adjusting for long-term tobacco use and other covariates, including childhood SES, family history of substance dependence, and long-term alcohol use, long-term cannabis use was robustly associated with hypomethylation of nine markers: cg05575921, cg21566642, cg03636183, cg21161138, cg01940273, cg17739917, cg05086879, cg02978227, cg23079012. Six of the nine cannabis-related markers were associated with gene expression levels, suggesting meaningful biological associations. Cannabis quitters showed less extreme DNA hypomethylation than long-term cannabis users. Long-term cannabis use could affect the epigenome similarly to tobacco use, possibly though smoke inhalation. Cannabis cessation, like tobacco cessation, may reverse altered DNA methylation.

